# Species Specificity in Major Urinary Proteins by Parallel Evolution

**DOI:** 10.1371/journal.pone.0003280

**Published:** 2008-09-25

**Authors:** Darren W. Logan, Tobias F. Marton, Lisa Stowers

**Affiliations:** Department of Cell Biology, The Scripps Research Institute, La Jolla, California, United States of America; The Rockefeller University, United States of America

## Abstract

Species-specific chemosignals, pheromones, regulate social behaviors such as aggression, mating, pup-suckling, territory establishment, and dominance. The identity of these cues remains mostly undetermined and few mammalian pheromones have been identified. Genetically-encoded pheromones are expected to exhibit several different mechanisms for coding 1) diversity, to enable the signaling of multiple behaviors, 2) dynamic regulation, to indicate age and dominance, and 3) species-specificity. Recently, the major urinary proteins (Mups) have been shown to function themselves as genetically-encoded pheromones to regulate species-specific behavior. Mups are multiple highly related proteins expressed in combinatorial patterns that differ between individuals, gender, and age; which are sufficient to fulfill the first two criteria. We have now characterized and fully annotated the mouse *Mup* gene content in detail. This has enabled us to further analyze the extent of Mup coding diversity and determine their potential to encode species-specific cues.

Our results show that the mouse *Mup* gene cluster is composed of two subgroups: an older, more divergent class of genes and pseudogenes, and a second class with high sequence identity formed by recent sequential duplications of a single gene/pseudogene pair. Previous work suggests that truncated *Mup* pseudogenes may encode a family of functional hexapeptides with the potential for pheromone activity. Sequence comparison, however, reveals that they have limited coding potential. Similar analyses of nine other completed genomes find *Mup* gene expansions in divergent lineages, including those of rat, horse and grey mouse lemur, occurring independently from a single ancestral *Mup* present in other placental mammals. Our findings illustrate that increasing genomic complexity of the *Mup* gene family is not evolutionarily isolated, but is instead a recurring mechanism of generating coding diversity consistent with a species-specific function in mammals.

## Introduction

Mouse major urinary proteins (Mups) are synthesized in the liver, secreted through the kidneys, and excreted in urine in milligram quantities per milliliter [1], [2]. This abundant protein excretion is thought to play a role in chemo-signaling between animals to coordinate social behavior. Mups belong to a large family of low-molecular weight ligand-binding proteins known as lipocalins, which share the fundamental tertiary structure of eight β-sheets arranged in a β-barrel open at one end with α-helices at both the N and C termini [3]. Consequently, they form a characteristic “glove” shape, encompassing a hydrophobic binding pocket that is able to bind specific small organic molecules [4].

The scope of function and mechanism of action of Mups remains controversial. A number of Mup small molecule ligands have been identified as male-specific volatile pheromones: molecular signals excreted by one individual that trigger an innate behavioral response in another member of the same species [5]. Mouse Mups have since been hypothesized to act as pheromone carrier proteins, which transport the volatile pheromones into the mucus filled pheromone detection organ; the vomeronasal organ (VNO). They have additionally been demonstrated to function as pheromone stabilizers in the environment, providing a slow release mechanism that extends the effective potency of these volatile molecules in male urine scent marks [6]. Finally, Mups have been shown to be a source of genetically encoded pheromones themselves [7]–[10]. However, the full extent of their function as species-specific pheromone signals has not been determined, largely because until recently the diversification of Mups in mouse was unclear.

Species-specific signals are expected to display several characteristics, including a mechanism for coding diversity to signal various social behaviors such as aggression, mating, pup-suckling, territory establishment, and dominance. Mups are known to be encoded by multiple paralogous genes, sufficient to fulfill this criteria [11]. Prior studies have identified individual *Mup* genes by comparing cloned DNA fragments with a number of expressed Mup protein and mRNA sequences [12]–[18]. Estimates based on hybridization to sequential genomic clones proposed that between 15 and 35 *Mup* genes and pseudogenes are clustered in a single locus on mouse chromosome 4 [11], [19]. Previous nomenclature classified the Mups into three groups, identifying an unknown number of highly similar *Mup* genes to comprise one group, potential pseudogenes in a second group, and more divergent *Mup* genes forming a third group [17], [19]. Despite these attempts to define the gene family, variation in intra-specific expression pattern, extremely high amino acid identity of expressed proteins, and a lack of nomenclature consistency has resulted in multiple *Mup* genes referred to by identical names in the Ensembl genome assembly [20]. The advance of genome sequencing has now enabled analysis and annotation of the genomic cluster. Recently, the mouse *Mup* gene cluster was partly characterized by manual genome annotation of a C57BL/6J genome assembly, identifying 19 predicted genes and 19 presumptive pseudogenes [21]. It has been hypothesized that the pseudogenes in the locus may in fact encode short, bio-active peptides that can themselves act as pheromones [9], [10], [14]. However, the coding potential of the pseudogene repertoire has not been evaluated.

Species-specific signals would additionally be expected to display dynamic regulation so that dominant and sub-ordinate males, females, and juveniles each excrete different signals to indicate their gender and status. Indeed, Mup expression is regulated by testosterone, thyroxine, and growth hormone with adult males having much higher Mup levels in urine than females or juveniles [1], [2]. Instead of expressing the entire repertoire of Mups, each individual expresses 4–12 of the proteins. This variable expression pattern has been hypothesized to create a protein “bar-code” defining individuality [16], [22]–[24]. Individual wild mice have unique expression patterns of Mups in their urine [24], [25]. Different lab strains each express different Mups, however individuals of the same strain express identical Mup repertoires as a result of inbreeding [16], [21]. *Mup* gene expression is therefore dynamically regulated by both genetic and endocrine mechanisms.

Lastly, we expect genetically encoded pheromones to generate signals that are species-specific so that ligands deposited in the environment do not lead to inappropriate behaviors such as aggression or mating between species. Species-specific Mup pheromones could evolve either by positive selective pressures acting on an existing *Mup* gene repertoire or by paralogous duplications of an ancestral *Mup*. Rats express a similar protein family, known as the α_2u_-globulins that share many of the same expression characteristics of the mouse Mups [11], [26]–[29]. Rat α_2u_-globulins are proposed to be encoded by an estimated 20 genes, are expressed dimorphically and combinatorially in urine and other exocrine glands, and the structure of a rat α_2u_-globulin shows striking homology to mouse Mups, including the ability to bind small hydrophobic molecules thought to be pheromones [30]–[32]. There is some evidence that rat α_2u_-globulins also function in intra-species communication by stimulating neurotransmitter release in the female amygdala and invoking locomotory behavior in a VNO-dependent manner [33]. Similar to the observation that mouse Mups carry activity independent of their ligand, it has been demonstrated that a recombinant rat α_2u_-globulin is sufficient to stimulate neuronal activation in the VNO [34]. Both the evolutionary relationship between mouse Mups and rat α_2u_-globulin and the extent to which they evolved in a species-specific manner is unknown.

Despite being the subject of intense study since their discovery over 45 years ago, the genomic locus of the *Mup* gene subfamily has yet to be fully investigated, and the phylogenetic relationships within and between species are unknown. Here, using known rodent Mup protein sequences to mine genome assemblies, we have characterized and annotated the *Mup* gene cluster in the mouse, and identified orthologous loci in a range of mammals, providing phylogenetic and structural evidence that *Mup* gene families show remarkable lineage specificity, consistent with a role in species-specific communication.

## Results

### Mouse Major Urinary Protein Gene Cluster

**Figure 1:**
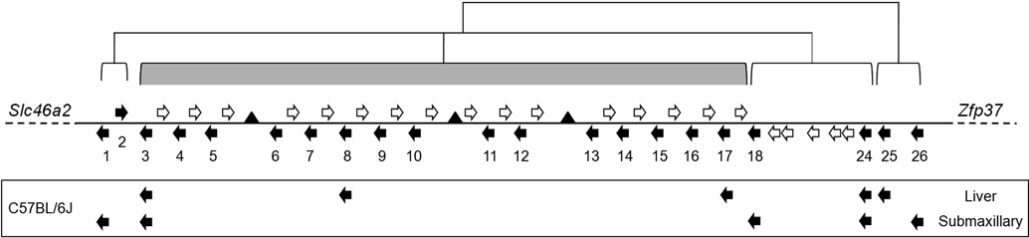
The mouse *Mup* gene cluster. Black arrows indicate direction of coding genes, numbered beneath, in the mouse genome. White arrows indicate direction of pseudogenes. Gaps in the genome are indicated by black triangles. The genes are arranged in two classes based on phylogeny, Class A in open brackets and Class B shaded grey. Genes expressed in male C57BL/6J liver and submaxillary glands indicated by black arrows, by RNA expression analysis.

The mouse *Mup* gene cluster is poorly annotated with repetitive nomenclature in the mouse genome sequence [20]. We first characterized the NCBI m37 C57BL/6J mouse genome assembly *Mup* loci, within a 1.92 Mb segment of chromosome 4 between *Slc46a2* and *Zfp37*, using a Hidden Markov Model of expressed rodent Mups. Our analysis identified 21 open reading frames (ORFs) encoding putative Mups, and a further 21 presumptive pseudogenes (fig. 1), 16 with insertions or deletions leading to a premature stop codon and 5 with the loss of an exon as a result of incomplete duplication. This is in agreement with a recent independent analysis [21]; however, we identified an additional two genes and two pseudogenes.

**Figure 2:**
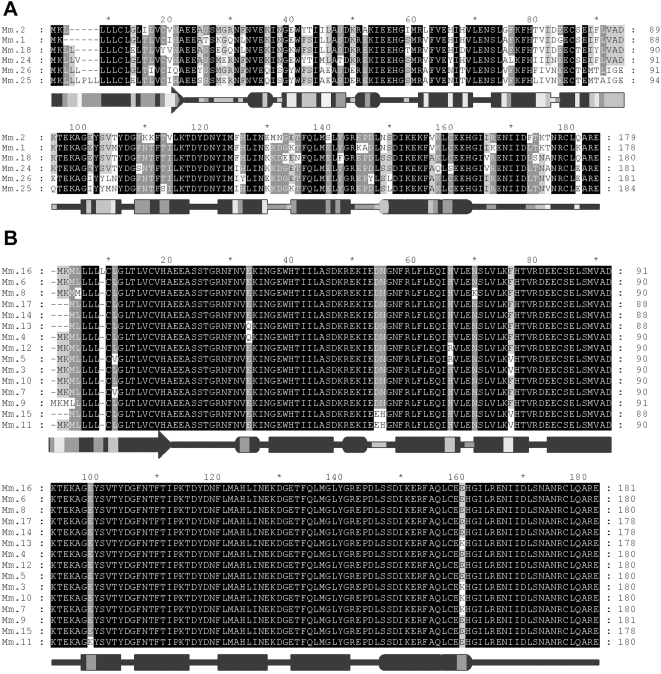
Homology within mouse Mup classes. A) Alignment of the predicted amino acid sequences of Class A Mups with the predicted secondary protein structure, shaded to indicate areas of least (dark) and most (light) variation within the sub-family. The arrow indicates the cleaved signal peptide, a rectangle indicates a β-sheet and an oval indicates a α-helix. B) Alignment of the predicted amino acid sequences of Class B Mups with the predicted secondary protein structure.

**Figure 3:**
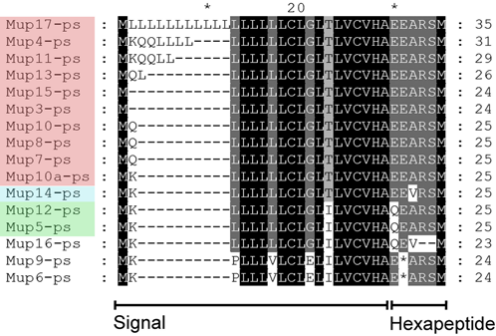
Mouse Class B *Mup* pseudogenes show limited coding potential. A) Alignment of the signal peptide plus hexapeptide sequences predicted by the mouse Class B pseudogenes. Stop codons (*) and gaps (-) are shown. The pseudogenes potentially encoding three distinct hexapeptide sequences are indicated by color. Three sequences (in white) have a disrupted hexapeptide sequence.

Identification of the repertoire of *Mup* genes next enabled us to categorize the family into two classes, Class A and B, based on sequence homology and genomic structure. Class A consists of 6 similar genes and 5 pseudogenes. The genes, *Mup1*, *Mup2*, *Mup18*, *Mup24*, *Mup25* and *Mup26* are 82–94% identical at the cDNA level and all but one (*Mup2*) is on the reverse strand (fig. 1, 2A). These are consistent with the “peripheral” gene regions described by Mudge et al. [21]. The remaining 15 highly similar *Mup* genes form Class B, all of which are greater than 97% identical at the cDNA level (fig. 2B). *Mup3* through *Mup17* are arranged sequentially on the reverse strand and encompass the formally classified “Group 1” genes and the *Mup* “central region” [19], [21]. The *Mup* pseudogenes have been proposed to encode bioactivity [9], [10], [14]. Therefore, we analyzed the pseudogene repertoire to determine if it displays hallmarks expected of pheromones. Our genomic analysis shows that each Class B gene is paired with a forward strand pseudogene in a divergent head-to-head manner (fig. 1). These pseudogenes all have a conserved G>T change in the first coding exon resulting in a premature stop. Others have hypothesized that these sequences may in fact encode a truncated protein consisting of a cleaved signal sequence followed by a functional hexapeptide (fig. 3A), and formally classified them as “Group 2” *Mup* genes [14]. Identification of the repertoire of genomic sequences enabled us to evaluate the ability of the pseudogenes to encode a pheromone family. When we aligned the 16 Class B pseudogenes we found only 3 distinct hexapeptide sequences in the cluster, which greatly limit their coding potential (fig. 3A).

### Origin of Class B Mups

The repetitive structure of Class B *Mup* genes and pseudogenes forming sequential blocks about 45 Kb in length has been previously described and proposed as the unit both of functional organization and evolution of the entire cluster [35]. However, greater percent identity of the genes within this class suggests they evolved more recently than the more divergent Class A genes (fig. 2). One Class A pair, *Mup1* and *Mup2*, is arranged in a head-to-head manner similar to the Class B *Mups*. We next determined whether this *Mup* gene pair provided the template for the successive duplications that resulted in Class B.

**Figure 4:**
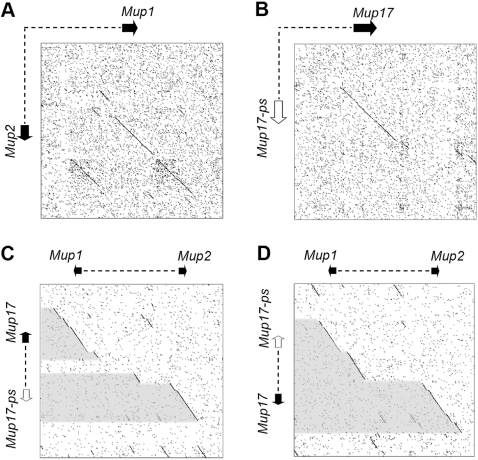
Class B *Mups* evolved from an inverted duplication of the *Mup1*, *Mup2* gene pair. Harr plot analyses of gene pairs using a sliding window of 9bp (A, B) and 11bp (C, D) with no mismatch. In all cases, the arrows represent genes (black) and pseudogenes (white) and are scaled to represent the distance between the ATG initiation codon and TGA termination codon. A) Analysis of the Class A *Mup1*, *Mup2* gene pair comparing 40 Kb of DNA in each direction from the midpoint between the genes. B) Analysis of the Class B *Mup17*, *Mup17-ps* gene/pseudogene pair comparing 30 Kb of DNA in each direction from the midpoint between the genes. C) Analysis of the Class A *Mup1*, *Mup2* gene pair with the Class B *Mup17*, *Mup17-ps* gene/pseudogene pair comparing 80 and 52 Kb of DNA respectively, spanning the gene pairs. D) As in C, except the comparison is inverted. This is the only comparison that shows homology (shaded) across all Class B genes and pseudogenes in addition to the entire intergenic spans (dashed lines).

Comparative analysis of the *Mup1* and inverted *Mup2* gene regions using Harr plots [36] shows a 25 Kb region spanning the genes that is duplicated and inverted (fig. 4A). In contrast, 28 Kb of the 43 Kb intergenic DNA is not duplicated. A similar comparison of the intergenic regions of Class B *Mup* pairs shows that each has an 11 Kb span, with 7.5 Kb of the 15 Kb intergenic distance not matching (fig. 4B shows *Mup17* and *Mup17 –ps* as an example). When we next compared the *Mup1*, *Mup2* pair with all Class B *Mup* pairs, we observed high sequence homology across the genes (fig. 4C). Interestingly, this did not extend through the Class B intergenic regions, as may be expected if the latter was a duplication of the former. However, when the sequence spanning *Mup1*, *Mup2* is compared with inverted Class B *Mup* pairs, there is near contiguous homology across both the Class B genes and the entire intergenic region (fig. 4D), suggesting that the latter is in fact an inverted duplication of the former.

**Figure 5:**
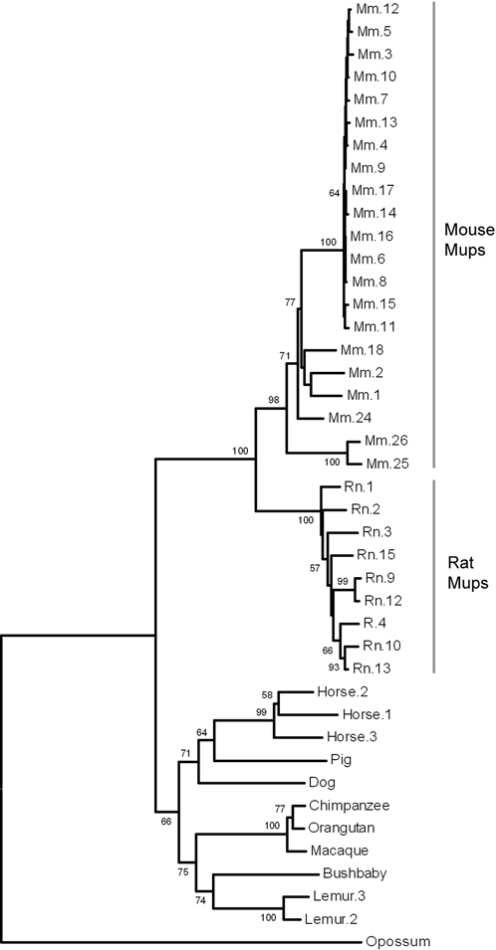
Phylogeny of Mup coding sequences in mammals. The predicted cDNA sequences were generated from open reading frames and aligned. The repeatability was tested by bootstrapping using 1000 replicates and a random seed. Interior branches with bootstrap support >50% are shown. The tree is rooted with a Mup-like cDNA previously reported in opossum [7].

The homology does not, however, extend contiguously across the *Mup1*, *Mup2* intergenetic region; there is a 25.5 Kb segment between *Mup1* and *Mup2* that has no homology between Class B. Since the cluster displays the hallmarks of significant dynamic instability, there may be additional modifications to the intergenic regions after the formation of the prototype Class B pair. We therefore searched for evidence betraying the origin of the non-homologous segment. We reasoned that if Class B *Mups* were generated from a Class A template, this segment must have inserted between *Mup1* and *Mup2* (or have been deleted between the prototype Class B gene/pseuodogene pair) subsequent to the original duplication. We found that the homology breakpoints correspond exactly with endogenous retroviral (ERV) long terminal repeat sequences (LTRs) (fig. S1) at both 5′ and 3′ ends. Moreover 89% of the intervening segment consists of interspersed repeats such as LINES, SINEs and LTRs, whereas the surrounding intergentic DNA contains just 41% (Class B) and 49% (*Mup1*, *Mup2*). It is therefore likely that the non-homologous segment of intervening DNA between *Mup1* and *Mup2* has a more recent origin than the rest of the intergenic region. This means that, when considered together with the phylogeny of the Mup cDNA sequences (fig. 5), Class A *Mups* are the ancestral genes and the canonical Class B *Mup* genes were generated from an inverted duplication of the ancestral *Mup1*, *Mup2* pair in the mouse lineage. The *Mup2* duplication resulted in a coding gene while the *Mup1* duplication pseudogenized. This gene/pseudogene pair then duplicated a number of times to form the Class B tandem array (fig. 1).

### Mup Gene Expression

The regulatory mechanisms that modulate the variable expression of Mups have not been identified; however identification of the genomic sequences that underlie expression in each strain is a first step towards elucidating regulation. We and others have identified specific Mup protein sequences excreted in the urine of inbred mice by a combination of western blot, isoelectric focusing, ion-exchange chromatography and electro-spray ionization mass spectrometry [7], [16], [21]. Minor differences of unclear significance have been previously observed, but our genomic analysis suggests that even single amino acid differences in protein sequences may reflect differences in gene expression, and thus have functional consequences. Therefore, to determine the genes that generate the transcriptional profile of *Mup* expression in the common mouse lab strain, C57BL/6J, we generated male liver and submaxillary gland cDNA before amplifying with *Mup*-specific PCR primers. We cloned and sequenced the resultant amplicons and compared them with the predicted gene sequences, previously published cDNA, and peptide sequences. We confirmed that male C57BL/6J mice express five distinct cDNA sequences in their liver, encoded by two Class A genes, *Mup24* and *Mup25*, and three Class B genes, *Mup3*, *Mup8* and *Mup17* (fig. 1). In addition to the male liver-expressed *Mups*, we can now identify the *Mup* genes expressed in C57BL/6J submaxillary glands: *Mup1* (previously reported as Mup IV), *Mup18* (previously reported as Mup V), *Mup24* and *Mup26* which are all members of the ancestral *Mup* gene subfamily, Class A. The only Class B gene product we identified from the submaxillary glands was *Mup3*.

### Independent Expansion of Rat Major Urinary Proteins

The rat α_2u_-globulins are encoded by an estimated 20 genes clustered on chromosome 5, as determined by Southern blot and fluorescence *in situ* hybridization [31], [37]. Like the mouse *Mup* genes, these rat genes are under multi-hormone regulation, are transcribed in the adult male liver and robustly expressed in urine, but are absent or barely detectable in the female and juvenile liver [28], [38].

**Figure 6:**
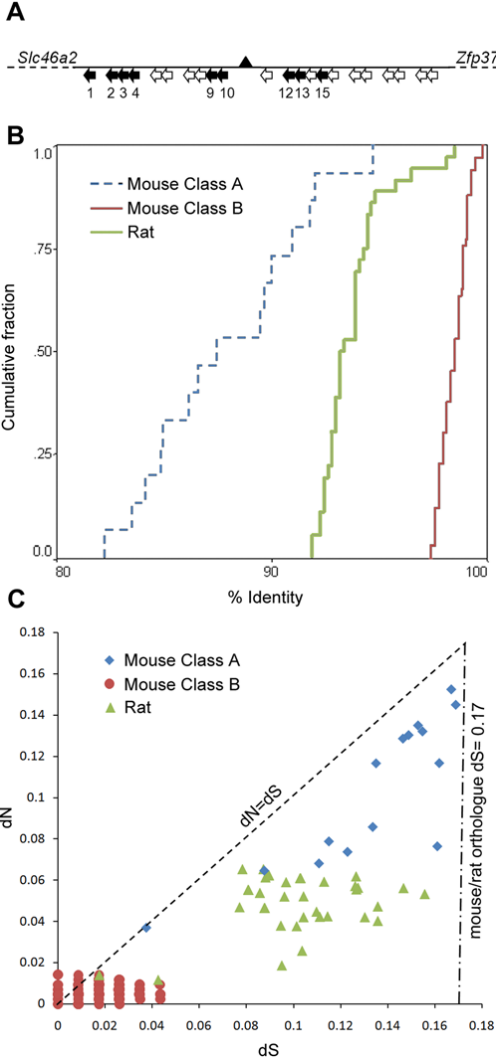
The rat *Mup* gene cluster differs in structure and divergence from the mouse. A) Black arrows indicate direction of coding genes, numbered beneath, in the rat *Mup* cluster. White arrows indicate direction of pseudogenes. Gaps in the genome alignment are indicated by black triangles. B) Cumulative fraction plot showing sequence variation within mouse Class A, Class B and rat Mup cDNA sequences. Each group differs significantly from the others (Kolmogorov-Smirnov test, P<1.3×10^−8^). C) Pair-wise synonymous substitution rates (dS) between mouse Class A (blue), Class B (red) and rat (green) Mup paralogues are all less than 0.171 (dot/dashed line), the calculated mean for mouse/rat orthologues [39]. When plotted against the non-synonymous substitution rates (dN), the Class A and rat Mups are beneath the line where dN = dS (dashed), indicating dN<dS for all pair-wise combinations of paralogues.

We identified the rat orthologues of mouse *Slc46a2* and *Zfp37* in the RGSC 3.4 brown rat, *Rattus norvegicus* genome assembly and analyzed the intervening 1.1 Mb region for rat genes homologous to those found in the mouse genome. We identified 9 ORFs and an additional 13 presumptive pseudogenes (fig. 6A) corresponding to the α_2u_-globulins and therefore may be considered rat *Mup* genes. Surprisingly, and in contrast to the mouse *Mup* cluster, the rat genes and pseudogenes are all arranged in a head-to-tail orientation on the reverse strand, there are no associated potential hexapeptide-encoding ORFs and they do not assort into two clearly distinct classes based on sequence similarity or structural arrangement. The range of sequence divergence in the rat *Mup* genes is instead intermediate to the two mouse classes, being 91–98% identical at the cDNA level (fig. 6B). There is also evidence that the rat cluster expanded in an alternative pair-wise manner, specifically rat *Mup9* and *Mup10*, *Mup12* and *Mup13*, and *Mup14-ps* and *Mup15* show conserved blocks of identity.

These differences may be explained by the *Mup* expansions having occurred at different periods during the evolutionary history of each lineage. We therefore carried out further analysis into whether the mouse and rat *Mup* gene repertoires expanded independently, after the rodent species diverged. In support of this, a phylogenetic reconstruction shows the mouse and rat predicted cDNAs segregate in distinct clades with strong bootstrap support (fig. 5). Rat and mouse-specific clades are also observed when a tree is reconstructed based only on synonymous substitutions (dS), which are considered to accumulate among gene lineages largely free from divergent selective pressures (fig. S2). Next we compared the relative dS accumulation within *Mups* of each species with a genome-wide estimate of divergence between mouse and rat. If the *Mup* repertoires were formed after the mouse/rat divergence, the dS accumulation would be expected to be less than 0.171, the calculated mean dS for orthologues formed by divergence [39]. For a conservative analysis we isolated the Class B from the recently formed Class A *Mups*, since high levels of gene conversion between paralogues result in artificially low rates of substitution (Class B dS = 0.0175, which is ten-fold lower than that seen in rat/mouse orthologues). However, even within the Class A and rat *Mup* paralogues, in which we find no evidence of recent gene conversion events, the dS values are lower than seen between rat/mouse orthologues (Table 1). These values are the mean for all paralogues, and are thus not reflective of the sequential nature of the duplication events. Therefore we also analyzed every pair-wise combination within Class A and found all had a dS<0.171 (fig. 6C), which implies that the paralogues formed post-speciation. In addition, all pair-wise comparisons within Class A and Rat *Mups* have a lower relative rate of non-synonymous substitutions than synonymous substitutions (dN<dS), which is consistent with a selective constraint acting on the genes (fig. 6C). Therefore, despite evidence for a conserved function, the inferred phylogeny, accumulation of synonymous substitutions and the differential organization of the *Mup* genomic loci all indicate that the mouse and rat gene lineages expanded independently, from one or a small number of ancestral *Mup* genes.

**Table 1:** Non-synonymous (dN) and synonymous (dS) substitution rates for all mouse Class A, Class B and rat *Mup* paralogues.

Genes	*dN*	*dS*	*dN/dS*
Mouse Class A	0.103	0.133	0.769
Mouse Class B	0.006	0.018	0.333
Rat	0.049	0.098	0.498

### Parallel Expansions of Non-Rodent Mup Clusters

Our finding that the last common ancestor of rat and mouse had either a single or small number of *Mups*, led us to determine the extent of *Mup* gene expansions across non-rodent lineages. Of the sequenced genomes available, we were able to identify orthologues of the *Slc46a2* and *Zfp37* genes and contiguous genomic sequence spanning the interval between the genes in nine additional placental mammals. We found that dog, pig, baboon, chimpanzee, bush-baby and orangutan each have a single *Mup* gene, with no evidence of additional pseudogenes, while humans have one presumptive pseudogene (caused by a G>A difference from the chimpanzee sequence that destroys a splice donor site). The *Mup* cluster in these species, as defined by the interval between neighboring genes, is 12–18 times smaller than mouse and 6–10 times smaller than rat, consistent with expansions in rodents (Table 2).

**Table 2:** The *Mup* gene cluster expanded at least four times in the mammalian lineage.

Binomial Name	Common Name	Chromo-some	Interval (Kb)^a^	Genes	Pseudo-genes	Total
*Mus musculus*	Mouse	4	1922	21	21	42
*Rattus norvegicus*	Rat	5	1068	9	13	22
*Equus caballus*	Horse	25	227	3	0	3
*Microcebus murinus*	Lemur	unassigned	120	2	1	3
*Pongo pygmaeus*	Orangutan	9	161	1	0	1
*Pan troglodytes*	Chimp	9	155	1	0	1
*Canis familiaris*	Dog	11	136	1	0	1
*Sus scrofa*	Pig	1	133	1	0	1
*Otolemur garnettii*	Bush baby	unassigned	112	1	0	1
*Macaca mulatta*	Macaque	15	110	1	0	1
*Homo sapiens*	Human	9	151	0	1	1

a The interval encompassing the *Mup* gene locus is defined here by the distance in Kb between the neighboring genes, *Slc46a2* and *Zfp3*.

Interestingly, two of the nine genomes did reveal further examples of lineage specific expansions. The horse (*Equus caballus*), has three *Mup* paralogues, arranged head-to-tail on the reverse strand of chromosome 25 (Table 2, fig. 5). The product of one of these has been previously isolated from dander and sublingual salivary glands. It was identified as a major horse allergen (accession: U70823), and has been used to detect additional expression in submaxillary glands and liver [40]. We also found that the grey mouse lemur (*Microcebus murinus*) has at least two *Mup* gene paralogues and one presumptive pseudogene (Table 2, fig. 5). These findings reinforce our conclusion that increasing genomic complexity of the *Mup* gene subfamily is not limited to rodents, but is instead a mechanism that has occurred multiple times in parallel in the mammalian lineage, consistent with a species-specific function.

We were unable to conclusively characterize *Mup* genes in any other placental mammalian genomes, largely because of limited sequencing coverage. The current genome alignments from cow and cat were not extensive enough to permit the analysis of a contiguous sequence spanning the entire interval, but we found single *Mups* linked to one of the adjacent genes. We also studied high coverage non-mammalian vertebrate genomes, including zebrafish, fugu and chicken, and found that the conserved syntenic block linking *Mups* with neighboring genes in placental mammals was disrupted. There is an independent expansion of 6 *Mup*-like genes in the marsupial opossum, *Monodelphis domestica*, yet because no conclusive syntenic relationship could be established and the sequences are sufficiently divergent from placental Mups, it remains possible that these are orthologous with another lipocalin subfamily [7].

## Discussion

### Mouse Mup Cluster

Our manual annotation of the *Mup* cluster in the NCBI m37 C57BL/6J mouse genome assembly identified 21 genes and 21 peudogenes, two more than a recent similar analysis that used a less complete assembly [21]. The additional genes reported here are *Mup10* and *Mup13*, both among the highly similar Class B *Mups*, and their associated pseudogenes. The current genome sequencing in the Class B region, while extensive, remains incomplete with three gaps found in the assembly (fig. 1). Given the highly repetitive nature of the Class B genes, we considered that these gaps may contain additional coding genes. The mean intergenic distance between each Class B coding gene is 77.2 Kb (+/− 2.9 SEM) and the gaps, of unknown sizes, are 60.5 Kb, 40.2 Kb and 6.2 Kb from the nearest adjacent genes. Indeed, we identified an additional unpaired pseudogene (*Mup10a –ps*) adjacent to one of these gaps, suggesting that at least one additional coding gene may be in the gap between *Mup10* and *Mup11*. Therefore, while we are confident the repertoire of Class A *Mups* is complete; there may be additional intervening Class B *Mup* genes and pseudogenes.

### Class B Structure and Function

The characterization of the *Mup* gene repertoire into two phylogenetically distinct subclasses, one older and one more recent, allowed us to determine the origin of the more recent expansion. We found that the Class A gene pair *Mup1* and *Mup2* provided the inverted template for the Class B genes and pseudogenes respectively. Murine endogenous retrovirus elements (ERV) are found localized with the Class B inverted duplication break points, and it has been proposed that recombination between nearby elements is the mechanism of duplication [21]. We have found ERV elements between and around the *Mup1* and *Mup2* genes, as would be expected if the Class B array originated from the inverted Class A pair through non-allelic homologous recombination. The multiple gene conversion events that likely took place during the evolution of the extremely repetitive mouse Class B array [12], [21] precludes an accurate estimation of the sequence by which the cluster expanded. However our findings imply that the full repertoire of Class B pseudogenes formed from an early pseudonization event, followed by duplication and gene conversion.

Others have proposed that these truncated, pseudogenized, *Mup* sequences may actually encode functional hexapeptides [14]. Non-synonymous/synonymous substitution analysis to determine whether the hexapeptide sequences were under selection proved inconclusive (not shown), because it was confounded by the short length of the hexapeptide-encoding DNA and the highly conserved nature of the sequences as a consequence of gene conversion. Having defined the repertoire of pseudogenes in the *Mup* cluster, we are now however able to evaluate the scope for the hexapeptide-encoding DNA to function as a family of pheromones. We found that their presence was limited to mice among the species we studied, and that their coding variation is extremely limited, providing at maximum three distinct signals. Experimental data has failed to find stable expression of hexapeptide mRNA in Mup-expressing tissues and no hexapeptides have been identified in urine [17].

### Mup Expansions Occurred in Species Specific Lineages

The phylogenetic reconstruction of the mouse and rat *Mup* gene clusters suggests independent expansion in each species (fig. 5, S2). While multiple gene conversion events can also result in the misleading appearance of a species-specific expansion, the more divergent Class A *Mups* form a distinct clade from the rat *Mups* and we find no evidence of gene conversion events in this class. Additionally, both mouse and rat *Mup* paralogues show lower rates of neutral substitution than would be expected between mouse/rat orthologues. Finally, others have observed fragments of a zinc-finger pseudogene repeated throughout the rat cluster [41]. These fragments appear to have duplicated in concert with the rat *Mups*, but are missing entirely in the mouse cluster. Taken together, and considered with the characteristic differences in the structure of the gene cluster in mouse and rat, these data strongly support parallel expansions in rodents. Moreover, our finding that similar, albeit more limited, *Mup* gene duplications have occurred in at least two more disparate mammalian lineages demonstrates the proclivity of *Mup* gene expansion in mammals.

Independent, post-speciation expansion is a characteristic found in other gene families involved in pheromone communication. The *androgen-binding protein* (*Abp*) gene family, which has been proposed to be a source of genetically encoded pheromones, has strikingly similar characteristics to that of *Mups*. They have undergone a large lineage-specific expansion in mouse since the divergence from rat, are arrayed in a cluster, and show parallel expansions in some additional mammalian species, but not others [42]–[44]. Both the V1R and V2R putative pheromone receptor gene families have been shown to have undergone lineage-specific expansions in mouse and rat [45]–[47]. Intriguingly, mouse and rat Mups specifically activate V2R expressing VNO neurons in their respective species, raising the possibility that *Mup* and V2R families co-evolved under species-specific positive selection [7], [34].

### Heterozygosity as Another Mechanism of Coding Diversity

The presence of a single protein in many species may appear to preclude a role in species-specific function due to a limitation in the amount of information that can be coded. Contrary to this, the single pig *Mup* gene encodes a salivary lipocalin (SAL, accession: NM_213814) that is dimorphically expressed in male submaxillary glands and binds known pig sex pheromones [48], [49]. Whether the protein itself has species-specific bioactivity is unknown, but interestingly two isoforms of SAL protein was isolated from a single male pig. The isoforms differ by 3 amino acids, and therefore may reflect heterozygosity, with significant genetic variation, at the single *Mup* gene. This also likely occurs in other species. For example, the previously reported horse Mup protein sequences are highly similar but not identical to those encoded in the sequenced horse genome [40], and there are significantly more mouse Mup proteins identified than is predicted in the mouse C57BL/6J genome, suggesting extensive heterozygosity in the wild mouse population [16], [24], [25], [50].

This additional level of variation may be maintained by balancing selection, thereby maximizing the coding potential of the *Mup* genes two-fold within any individual and permitting even single *Mup* genes to provide limited species-specific information. Diversity enhancing selection has been documented in other gene families, including those encoding hemoglobin and the major histocompatibility complex [51], [52]. Moreover, as chemosignals, Mups have been shown to influence social behavior on direct detection [7], [8], [10]. Therefore, an increase in coding potential could provide a distinct heterozygote advantage in successful mate choice or kin recognition [53], [54], both factors that would select for the maintenance of *Mup* heterozygosity in outbred populations.

### Ethological Role of Mups in Rodents

The ongoing sequencing of a number of rodent genomes will eventually provide further insight into the extent of *Mup* gene expansions in rodents. The species-specific behaviors that Mups have a role in, such as inter-male aggression and inbreeding avoidance, are not unique to rats and mice [55]–[57]. Therefore it will prove informative to determine whether *Mup* diversity is a common feature in rodent genomes, or whether the expansion seen in mouse and rat is anomalous.

Interestingly, males from other *Mus* species, including *Mus macedonius* and *Mus spretus*, appear to express either one or small number of Mups in their urine and these are largely invariant between individuals [58]. These mouse species live sympatrically with *Mus musculus domesticus* but their ecological niche is largely independent of humans and thus they have much lower population densities than the domestic mouse species. It has been suggested that *Mup* expansion occurred specifically in rodent species that live in densely populated, spatially overlapping social groups in close proximity to humans [59]. This environment, common to both domestic mice and brown rats, requires a robust mechanism for species-specific social behavior. Further genome sequencing will enable us to determine whether these differences are reflected in a smaller *Mup* gene repertoire in *Mus macedonius* and *Mus spretus*, or simply due to a reduction in gene expression.

## Materials and Methods

### Genome Analyses

We used all known mouse Mup protein sequences as queries to BLAST against the NCBI m37 C57BL/6J mouse (*Mus musculus*) genome assembly. This identified the genomic location of the *Mup* gene cluster in a 1.9 Mb interval between genes *Slc46a2* (accession: NM_021053) and *Zfp37* (accession: NM_009554) and ruled out the existence of additional *Mup* loci. We then exported and annotated the position of candidate genes in the intervening sequence using a Hidden Markov Model (HMM) based on the known protein sequences. The sequence spanning each HMM hit, plus 10 Kb of neighboring sequence, was then exported and individual mouse Mup protein sequences were used to conduct protein-to-genomic sequence alignments with GeneWise (http://www.ebi.ac.uk/wise2/), a tool used widely in gene prediction and genome annotation [60]. Because the open reading frames determined by GeneWise were extremely highly conserved in coding sequence, surrounding non-coding sequence and gene structure, we are confident that all genes in the exported sequence were correctly identified. However, after characterizing all *Mup* sequences, we incorporated them into further HMMs and re-annotated the interval. No further genes or pseudogenes were found.

We then identified orthologues of *Slc46a2* and *Zfp37* in other species and repeated this analysis of the syntenic interval using the following genome sequence assemblies, all obtained from Ensembl (http://www.ensembl.org): rat (*Rattus norvegicus*, RGSC 3.4), human (*Homo sapiens*, NCBI 36), chimpanzee (*Pan troglodytes*, CHIMP2.1), dog (*Canis familiaris*, Canfam 2.0), cow (*Bos Taurus*, Btau 3.1), chicken (*Gallus gallus*, WASHUC2), cat (*Felis catus*, CAT), horse (*Equus caballus*, EquCab2), mouse lemur (*Microcebus murinus*, micMur1), orangutan (*Pongo pygmaeus abelii*, PPYG2), pig (*Sus scrofa*, Sscrofa1), bushbaby (*Otolemur garnettii*, otoGar1) and macaque (*Macaca mulatta*, MMUL 1.0) [20]. In each case the genes in these regions were resolved using iterative HMMs generated from known sequences and those subsequently characterized. No additional *Mup* sequences were found at other loci. All other placental species with genomic sequence data had insufficient coverage at the time of analysis, and all other species genomes had disrupted synteny and no highly homologous *Mup* genes.

### Evolutionary Analyses

The deduced cDNA and peptide sequences of Mups were aligned using ClustalW2 [61]. GeneDoc (http://www.nrbsc.org/gfx/genedoc/) was used to visualize the alignments and calculate the cumulative fraction plots of DNA sequence variation. Secondary structure was calculated using the PSIPRED prediction method [62]. Synonymous/non-synonymous substitutions were calculated using SNAP (http://www.hiv.lanl.gov), based on the methods of Nei and Gojobori [63]. Phylogenetic trees were reconstructed using MEGA3 [64], from aligned cDNA sequences using the neighbor-joining method with the Kimura-2 parameter model of substitution [65]. The repeatability of the tree was evaluated using the bootstrap method with 1000 pseudo-replications. Gaps in the alignment were not used in the reconstruction. Other methods (including UPGMA and minimum evolution) and models (including p-distance, number of differences and Tajima-Nei models) of phylogenetic reconstruction resulted in differences in arrangement only within the highly similar Class B *Mup* genes. Similarly, phylogenetic reconstructions using predicted amino acid sequences, synonymous and non-synonymous sites recapitulated the cDNA based reconstruction; therefore we are confident the phylogeny is robust.

### Locus Structure

Harr plot analysis [36] was carried out on mouse genomic DNA sequences using the DNAdot tool (http://www.vivo.colostate.edu/molkit/dnadot/). A sliding window of 9 base pairs was used to determine identity in analyses between genes, and a sliding window of 11 base pairs was used to compare gene pairs. In both cases high stringencies were used, with no mismatch permitted. Intergenic retroviral elements were identified using RepeatMasker Open-3.2.3 (http://www.repeatmasker.org/).

### Mup Expression

Sets of degenerate oligonucleotide primers were synthesized complementary to the entire mouse Class A and Class B *Mup* repertoire. The forward primer sequences are (5′ to 3′), Mup1: ATGAAGCTGCTGCTGTGT, Mup2: ATGAAGCTGCTGCTGCTGT, Mup3–7,9–10,12,16: ATGAAGATGCTGCTGCTG, Mup8: ATGAAGATGATGCTGCTG, Mup11: ATGAAGATGCTGTTGCTG, Mup13–14,17: ATGCTGTTGCTGCTGTGT, Mup15: ATGCTGCTGCTGCTGTGT, Mup18: ATGAAGCTGTTGCTGCTG, Mup24: ATGAAGCTGCTGGTGCTG, Mup25: ATGAAGCTGCTGCTGCCG, Mup26: ATGAAGCTGTTGCTGCTG. The reverse primers are, Mup1: TCATTCTCGGGCCTTGAG, Mup2–18: TCATTCTCGGGCCTGGAG, Mup24: TCATTCTCGGGCCTCAAG, Mup25–26: TCATTCTCGGGCCTCGAG. RNA was extracted from the liver and submaxillary glands of two male C57BL/6J mice, using an RNeasy extraction kit (Qiagen, Valencia, USA) and reverse transcribed using an oligo-dT primer and SuperScript II reverse transcriptase (Invitrogen, Carlsbad, USA). Polymerase chain reaction amplicons were cloned into pCRII-TOPO (Invitrogen, Carlsbad, USA) and sequenced. The resultant sequences were then aligned with the predicted cDNA sequences of the *Mup* gene repertoire.

### Database Submission

Nucleotide sequence data reported are available in the DDBJ/EMBL/GenBank databases under the accession numbers: EU882229 - EU882236, and in the Third Party Annotation Section of the DDBJ/EMBL/GenBank databases under the accession numbers TPA: BK006638 – BK006679.

## Supporting Information

Figure S1 Detail of homology between Mup1, Mup2 and Class B pairs. The intergenic region between mouse Mup1 and Mup2 (top, black arrows) is homologous with the intergenic regions between Class B pseudogenes (bottom, white arrow) and genes (black arrow). A large break in the homology in the Mup1, Mup2 intergenic region (red) is likely due to a more recent endogenous retroviral mediated insertion, as ERV long terminal repeats are found across the homology break points (green).
(0.21 MB TIF)

Figure S2 Analysis of synonymous sequence divergence between mouse and rat Mups. An unrooted tree reconstructed from a codon-based likelihood analysis of synonymous substitutions between mouse Class A (blue), Class B (red) and rat (green) coding sequences. Branch lengths are in units of synonymous substitutions per synonymous site.
(0.26 MB TIF)

## References

[1] Finlayson JS, Asofsky R, Potter M, Runner CC (1965). Major urinary protein complex of normal mice: origin.. Science.

[2] Szoka PR, Paigen K (1978). Regulation of mouse major urinary protein production by the Mup-A gene.. Genetics.

[3] Ganfornina MD, Gutierrez G, Bastiani M, Sanchez D (2000). A phylogenetic analysis of the lipocalin protein family.. Mol Biol Evol.

[4] Flower DR (1996). The lipocalin protein family: structure and function.. Biochem J.

[5] Stowers L, Marton T (2005). What is a pheromone? Mammalian pheromones reconsidered.. Neuron.

[6] Hurst JL, Robertson DHL, Tolladay U, Beynon RJ (1998). Proteins in urine scent marks of male house mice extend the longevity of olfactory signals.. Anim Behav.

[7] Chamero P, Marton TF, Logan DW, Flanagan K, Cruz JR (2007). Identification of protein pheromones that promote aggressive behaviour.. Nature.

[8] Marchlewska-Koj A, Cavaggioni A, Mucignat-Caretta C, Olejnicz P (2000). Stimulation of estrus in female mice by male urinary proteins.. J Chem Ecol.

[9] More L (2006). Mouse major urinary proteins trigger ovulation via the vomeronasal organ.. Chem Senses.

[10] Mucignat-Caretta C, Caretta A, Cavaggioni A (1995). Acceleration of puberty onset in female mice by male urinary proteins.. J Physiol.

[11] Hastie ND, Held WA, Toole JJ (1979). Multiple genes coding for the androgen-regulated major urinary proteins of the mouse.. Cell.

[12] Clark AJ, Chave-Cox A, Ma X, Bishop JO (1985). Analysis of mouse major urinary protein genes: variation between the exonic sequences of group 1 genes and a comparison with an active gene out with group 1 both suggest that gene conversion has occurred between MUP genes.. EMBO J.

[13] Clark AJ, Clissold PM, Bishop JO (1982). Variation between mouse major urinary protein genes isolated from a single inbred line.. Gene.

[14] Clark AJ, Ghazal P, Bingham RW, Barrett D, Bishop JO (1985). Sequence structures of a mouse major urinary protein gene and pseudogene compared.. EMBO J.

[15] Kuhn NJ, Woodworth-Gutai M, Gross KW, Held WA (1984). Subfamilies of the mouse major urinary protein (MUP) multi-gene family: sequence analysis of cDNA clones and differential regulation in the liver.. Nucleic Acids Res.

[16] Robertson DH, Cox KA, Gaskell SJ, Evershed RP, Beynon RJ (1996). Molecular heterogeneity in the Major Urinary Proteins of the house mouse Mus musculus.. Biochem J.

[17] Shahan K, Denaro M, Gilmartin M, Shi Y, Derman E (1987). Expression of six mouse major urinary protein genes in the mammary, parotid, sublingual, submaxillary, and lachrymal glands and in the liver.. Mol Cell Biol.

[18] Shahan K, Gilmartin M, Derman E (1987). Nucleotide sequences of liver, lachrymal, and submaxillary gland mouse major urinary protein mRNAs: mosaic structure and construction of panels of gene-specific synthetic oligonucleotide probes.. Mol Cell Biol.

[19] Bishop JO, Clark AJ, Clissold PM, Hainey S, Francke U (1982). Two main groups of mouse major urinary protein genes, both largely located on chromosome 4.. Embo J.

[20] Hubbard TJ, Aken BL, Beal K, Ballester B, Caccamo M (2007). Ensembl 2007.. Nucleic Acids Res.

[21] Mudge JM, Armstrong SD, McLaren K, Beynon RJ, Hurst JL (2008). Dynamic instability of the major urinary protein gene family revealed by genomic and phenotypic comparisons between C57 and 129 strain mice.. Genome Biol.

[22] Armstrong SD, Robertson DH, Cheetham SA, Hurst JL, Beynon RJ (2005). Structural and functional differences in isoforms of mouse major urinary proteins: a male-specific protein that preferentially binds a male pheromone.. Biochem J.

[23] Cheetham SA, Thom MD, Jury F, Ollier WE, Beynon RJ (2007). The genetic basis of individual recognition signals in the mouse.. Curr Biol.

[24] Beynon RJ, Hurst JL (2003). Multiple roles of major urinary proteins in the house mouse, Mus domesticus.. Biochem Soc Trans.

[25] Robertson DH, Hurst JL, Bolgar MS, Gaskell SJ, Beynon RJ (1997). Molecular heterogeneity of urinary proteins in wild house mouse populations.. Rapid Commun Mass Spectrom.

[26] Roy AK, Neuhaus OW (1966). Proof of the hepatic synthesis of a sex-dependent protein in the rat.. Biochim Biophys Acta.

[27] Roy AK, Neuhaus OW (1966). Identification of rat urinary proteins by zone and immunoelectrophoresis.. Proc Soc Exp Biol Med.

[28] Roy AK, Neuhaus OW (1967). Androgenic control of a sex-dependent protein in the rat.. Nature.

[29] Roy AK, Neuhaus OW, Harmison CR (1966). Preparation and characterization of a sex-dependent rat urinary protein.. Biochim Biophys Acta.

[30] Bocskei Z, Groom CR, Flower DR, Wright CE, Phillips SE (1992). Pheromone binding to two rodent urinary proteins revealed by X-ray crystallography.. Nature.

[31] Kurtz DT (1981). Rat alpha 2u globulin is encoded by a multigene family.. J Mol Appl Genet.

[32] MacInnes JI, Nozik ES, Kurtz DT (1986). Tissue-specific expression of the rat alpha 2u globulin gene family.. Mol Cell Biol.

[33] Mucignat-Caretta C, Colivicchi MA, Fattori M, Ballini C, Bianchi L (2006). Species-specific chemosignals evoke delayed excitation of the vomeronasal amygdala in freely-moving female rats.. J Neurochem.

[34] Krieger J, Schmitt A, Lobel D, Gudermann T, Schultz G (1999). Selective activation of G protein subtypes in the vomeronasal organ upon stimulation with urine-derived compounds.. J Biol Chem.

[35] Clark AJ, Hickman J, Bishop J (1984). A 45-kb DNA domain with two divergently orientated genes is the unit of organisation of the murine major urinary protein genes.. Embo J.

[36] Harr P, Hagblom P, Gustafsson P (1982). Two-dimensional graphic analysis of DNA sequence homologies.. Nucleic Acids Res.

[37] McFadyen DA, Addison W, Locke J (1999). Genomic organization of the rat alpha 2u-globulin gene cluster.. Mamm Genome.

[38] Kurtz DT, Feigelson P (1977). Multihormonal induction of hepatic alpha2u-globulin mRNA as measured by hybridization to complementary DNA.. Proc Natl Acad Sci U S A.

[39] Wang W, Zheng H, Yang S, Yu H, Li J (2005). Origin and evolution of new exons in rodents.. Genome Res.

[40] Gregoire C, Rosinski-Chupin I, Rabillon J, Alzari PM, David B (1996). cDNA cloning and sequencing reveal the major horse allergen Equ c1 to be a glycoprotein member of the lipocalin superfamily.. J Biol Chem.

[41] Emes RD, Beatson SA, Ponting CP, Goodstadt L (2004). Evolution and comparative genomics of odorant- and pheromone-associated genes in rodents.. Genome Res.

[42] Emes RD, Riley MC, Laukaitis CM, Goodstadt L, Karn RC (2004). Comparative evolutionary genomics of androgen-binding protein genes.. Genome Res.

[43] Laukaitis CM, Dlouhy SR, Emes RD, Ponting CP, Karn RC (2005). Diverse spatial, temporal, and sexual expression of recently duplicated androgen-binding protein genes in Mus musculus.. BMC Evol Biol.

[44] Laukaitis CM, Heger A, Blakley TD, Munclinger P, Ponting CP (2008). Rapid bursts of androgen binding protein (Abp) gene duplication occurred independently in diverse mammals.. BMC Evol Biol.

[45] Lane RP, Young J, Newman T, Trask BJ (2004). Species specificity in rodent pheromone receptor repertoires.. Genome Res.

[46] Shi P, Bielawski JP, Yang H, Zhang YP (2005). Adaptive diversification of vomeronasal receptor 1 genes in rodents.. J Mol Evol.

[47] Yang H, Shi P, Zhang YP, Zhang J (2005). Composition and evolution of the V2r vomeronasal receptor gene repertoire in mice and rats.. Genomics.

[48] Loebel D, Scaloni A, Paolini S, Fini C, Ferrara L (2000). Cloning, post-translational modifications, heterologous expression and ligand-binding of boar salivary lipocalin.. Biochem J 350 Pt.

[49] Marchese S, Pes D, Scaloni A, Carbone V, Pelosi P (1998). Lipocalins of boar salivary glands binding odours and pheromones.. Eur J Biochem.

[50] Beynon RJ, Veggerby C, Payne CE, Robertson DH, Gaskell SJ (2002). Polymorphism in major urinary proteins: molecular heterogeneity in a wild mouse population.. J Chem Ecol.

[51] Bernatchez L, Landry C (2003). MHC studies in nonmodel vertebrates: what have we learned about natural selection in 15 years?. J Evol Biol.

[52] Storz JF, Baze M, Waite JL, Hoffmann FG, Opazo JC (2007). Complex signatures of selection and gene conversion in the duplicated globin genes of house mice.. Genetics.

[53] Sherborne AL, Thom MD, Paterson S, Jury F, Ollier WE (2007). The genetic basis of inbreeding avoidance in house mice.. Curr Biol.

[54] Thom MD, Stockley P, Jury F, Ollier WE, Beynon RJ (2008). The direct assessment of genetic heterozygosity through scent in the mouse.. Curr Biol.

[55] Payne AP (1973). A comparison of the aggressive behaviour of isolated intact and castrated male golden hamsters towards intruders introduced into the home cage.. Physiol Behav.

[56] Demas GE, Moffatt CA, Drazen DL, Nelson RJ (1999). Castration does not inhibit aggressive behavior in adult male prairie voles (Microtus ochrogaster).. Physiol Behav.

[57] Kruczek M (2007). Recognition of kin in bank voles (Clethrionomys glareolus).. Physiol Behav.

[58] Robertson DH, Hurst JL, Searle JB, Gunduz I, Beynon RJ (2007). Characterization and comparison of major urinary proteins from the house mouse, Mus musculus domesticus, and the aboriginal mouse, Mus macedonicus.. J Chem Ecol.

[59] Beynon RJ, Hurst JL, Turton MJ, Robertson DHL, Armstrong SD, Hurst JL, Beynon RJ, Roberts SC, Wyatt T (2008). Urinary lipocalins in Rodenta: Is there a generic model.. Chemical Signals in Vertebrates.

[60] Birney E, Clamp M, Durbin R (2004). GeneWise and Genomewise.. Genome Res.

[61] Larkin MA, Blackshields G, Brown NP, Chenna R, McGettigan PA (2007). Clustal W and Clustal X version 2.0.. Bioinformatics.

[62] Jones DT (1999). Protein secondary structure prediction based on position-specific scoring matrices.. J Mol Biol.

[63] Nei M, Gojobori T (1986). Simple methods for estimating the numbers of synonymous and nonsynonymous nucleotide substitutions.. Mol Biol Evol.

[64] Kumar S, Tamura K, Nei M (2004). MEGA3: Integrated software for Molecular Evolutionary Genetics Analysis and sequence alignment.. Brief Bioinform.

[65] Kimura M (1980). A simple method for estimating evolutionary rates of base substitutions through comparative studies of nucleotide sequences.. J Mol Evol.

