# Supplementary figures and images for: Species Specificity in Major Urinary Proteins by Parallel Evolution
Source: PLoS One. 2008 Sep 25;3(9):e3280. doi: 10.1371/journal.pone.0003280 (PMC2533699; doi:10.1371/journal.pone.0003280)

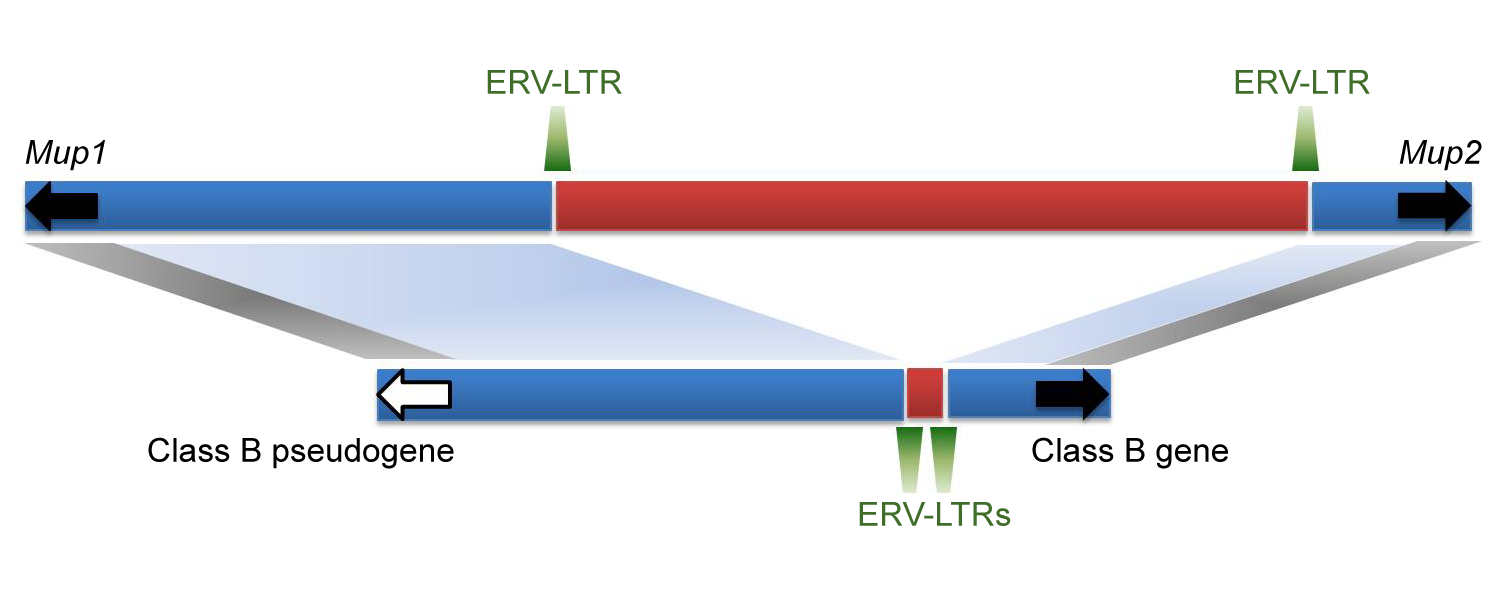

Supplement: Figure S1 — Detail of homology between Mup1, Mup2 and Class B pairs. The intergenic region between mouse Mup1 and Mup2 (top, black arrows) is homologous with the intergenic regions between Class B pseudogenes (bottom, white arrow) and genes (black arrow). A large break in the homology in the Mup1, Mup2 intergenic region (red) is likely due to a more recent endogenous retroviral mediated insertion, as ERV long terminal repeats are found across the homology break points (green). (0.21 MB TIF) [file pone.0003280.s001.tif]

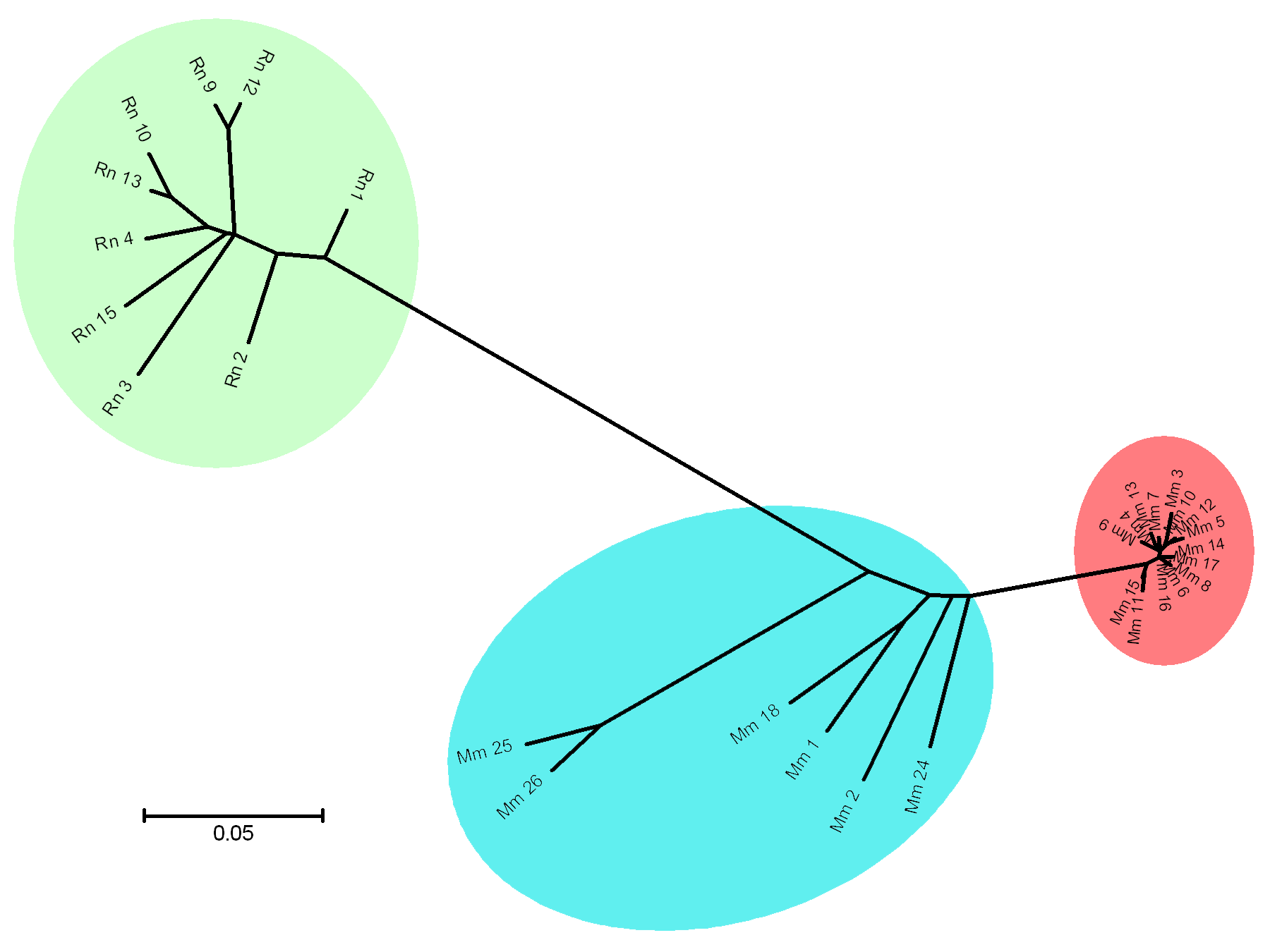

Supplement: Figure S2 — Analysis of synonymous sequence divergence between mouse and rat Mups. An unrooted tree reconstructed from a codon-based likelihood analysis of synonymous substitutions between mouse Class A (blue), Class B (red) and rat (green) coding sequences. Branch lengths are in units of synonymous substitutions per synonymous site. (0.26 MB TIF) [file pone.0003280.s002.tif]
